# A Nonlinear Framework of Delayed Particle Smoothing Method for Vehicle Localization under Non-Gaussian Environment

**DOI:** 10.3390/s16050692

**Published:** 2016-05-13

**Authors:** Zhu Xiao, Vincent Havyarimana, Tong Li, Dong Wang

**Affiliations:** 1 College of Computer Science and Electronic Engineering, Hunan University, Changsha 410082, China; zhxiao@hnu.edu.cn (Z.X.); havincent14@hnu.edu.cn (V.H.); litong@hnu.edu.cn (T.L.); 2 State Key Laboratory of Integrated Service Networks, Xidian University, Xi’an 710071, China; 3 Department of Applied Sciences, Ecole Normale Supérieure, 6983 Bujumbura, Burundi

**Keywords:** particle filter, fixed-delay smoothing, non-Gaussian noise, Ensemble Kalman Filter, vehicle localization

## Abstract

In this paper, a novel nonlinear framework of smoothing method, non-Gaussian delayed particle smoother (nGDPS), is proposed, which enables vehicle state estimation (VSE) with high accuracy taking into account the non-Gaussianity of the measurement and process noises. Within the proposed method, the multivariate Student’s *t*-distribution is adopted in order to compute the probability distribution function (PDF) related to the process and measurement noises, which are assumed to be non-Gaussian distributed. A computation approach based on Ensemble Kalman Filter (EnKF) is designed to cope with the mean and the covariance matrix of the proposal non-Gaussian distribution. A delayed Gibbs sampling algorithm, which incorporates smoothing of the sampled trajectories over a fixed-delay, is proposed to deal with the sample degeneracy of particles. The performance is investigated based on the real-world data, which is collected by low-cost on-board vehicle sensors. The comparison study based on the real-world experiments and the statistical analysis demonstrates that the proposed nGDPS has significant improvement on the vehicle state accuracy and outperforms the existing filtering and smoothing methods.

## 1. Introduction

Nowadays, vehicle localization is one of the most fundamental challenges of intelligent transport systems (ITS) where many researchers and practitioners are widely interested in improving the performance of the vehicle positioning systems [1,2]. To improve the vehicle state information, nonlinear filtering methods are employed in [3], which are adequate for handling the nonlinearity by considering that the system may be corrupted by zero-mean Gaussian white sequences. Although the choice of this assumption is usually justified by the law of large numbers [4], it is rather motivated in more cases by the fact that the normal distributions are mathematically convenient and makes the Gaussian models easier to handle than non-Gaussian models. However, there is evidence that in several engineering systems used to solve nonlinear state estimation problems, noises generally follow a non-Gaussian distribution. This observation is supported in [5,6,7,8,9,10,11], which demonstrated that many noisy environments can be addressed more accurately as non-Gaussian rather than Gaussian model. In the literature, Gaussian sum particle filtering (GSPF) method built from banks of Gaussian particle filters (GPFs) [12] has been widely used not only to overcome degeneracy in particle filter (PF) but also because it has good performance for the state estimation in non-Gaussian environment [8,13,14] and for transportation systems [15,16]. The filtering methods are in general recursive algorithms based on the conditional expectation of the state given all states and measurements up to the current time step. In recent years, smoothing methods, which estimate the states by using available measurements in the future and are used in order to improve filtering problems, have gained more consideration in the literature [17,18,19]. Especially based on particle theory (*i.e.*, particle smoothing), smoothing methods find their applications in guidance systems, integrated inertial navigation and passive sensor based target tracking [20,21,22] and are used in order to improve filtering problems [23]. Authors in [21] have used Rao-Blackwellized particle smoother (RBPS) for jointly estimating the position of the robot and the map. Recall that the Rao-Blackwellization takes advantage of a linear substructure in the model, which can be modeled by a Kalman filter (KF).

The main objective of this paper is to develop a nonlinear framework of smoothing technique that enables vehicle localization and state estimation with high accuracy taking into account the non-Gaussianity of the measurement and process noises. To achieve this, a new approach named non-Gaussian delayed particle smoother (nGDPS) for vehicle localization is proposed. The multivariate Student’s *t*-distribution is adopted in order to compute the probability distribution function (PDF) related to the process and measurement noises, which are assumed to be non-Gaussian distributed. We consider the Student’s *t*-distribution to investigate how the noise can influence on the improvement of estimation performance by varying the values of degree of freedom. Within the proposed nGDPS, the optimal estimator is designed based on fixed-delay smoothing (FDS) technique and the Ensemble KF (EnKF) [24] in order to improve the existing particle filter (PF). Its key advantage is to attain the improved estimates and lower error covariance and thus provide the accurate measurement information. For the computation of distribution parameters for nGDPS, an EnKF based approach is designed to cope with the mean and the covariance matrix of the proposal non-Gaussian distribution [25]. Moreover, we design a delayed Gibbs sampling algorithm that incorporates smoothing of the sampled trajectories over a fixed delay. The vehicle localization accuracy can be enhanced by Gibbs sampling method since it avoids the weight degeneracy problem during the particle smoothing.

To validate its performance, the proposed method is applied to a vehicle localization and state estimation problem. We evaluate its estimation improvement based on the non-Gaussianity of the noises [26], by introducing the so-called Gaussian delayed particle smoother (GDPS) where process and measurement noises are assumed to be Gaussian distributed. The Gaussian distribution is gotten from the multivariate-distribution [27], since the value of degrees of freedom increases; the univariate *t*-distribution approaches the Gaussian distribution [28]. Moreover, we compare nGDPS with the existing nonlinear filtering methods such as the standard PF and Gaussian sum particle filter (GSPF) and the existing nonlinear smoothing methods such as Rao-Blackwellized particle smoother (RBPS), fixed-point particle smoothing (FPPS) and the fixed-interval particle smoothing (FIPS). The assessment of the comparison study is operated using real vehicle information collected in urban environment. Instead of using expensive sensors [3], low-cost GPS receiver and on-board vehicle sensors including the wheel speed and acceleration sensors are used to provide the vehicle information such as position, velocity, and acceleration respectively. The experiments and the statistical analysis (in terms of root mean square error (RMSE)) demonstrate that the proposed approach has significant improvement on the vehicle state accuracy and outperforms the aforementioned filtering and smoothing methods.

The remainder of this paper is organized as follows. Section 2 describes the problem formulation. Section 3 is devoted to the non-Gaussian delayed particle smoothing (nGDPS) method. The computation of the proposal distribution parameters for nGDPS and the delayed Gibbs sampling method for degeneracy problem are also presented. Section 4 highlights the nGDPS algorithm for vehicle state estimation. Section 5 presents the experimental results. Finally, Section 6 concludes the paper.

## 2. Problem Formulation

The proposed approach is assumed to follow the nonlinear non-Gaussian model and is given by the following system equations:

(1) $$\left(S\right)\equiv \left\{ \begin{array}{l} X_{k}=f_{k}\left(X_{k-1},\phi_{k-1}\right)\\ Y_{k}=h_{k}\left(X_{k},\gamma_{k}\right) \end{array}\right.$$

where $\left\{X_{k};X\in IR^{m_{X}};m,k\in IN\right\}$ represents the state vector where $m_{X}$ is the dimension size of the state vector. The measurements $\left\{Y_{k};Y\in IR^{m_{Y}};m,k\in IN\right\}$ are conditionally independent given $\left\{X_{k},k\in IN\right\}$ and are expressed by the distribution $p\left(Y_{k}|X_{k}\right)$. Here, $m_{Y}$ is the dimension size of the measurement vector. The matrices $f_{k}$ and $h_{k}$ stand for the process and measurement model functions.

Moreover, $\phi_{k-1}$ and $\gamma_{k}$ indicate the process noise and measurement noise sequences, respectively. These noises are assumed to be non-Gaussian distributed. Among other distributions for computing the probability density function (PDF) associated to the non-Gaussian noise [5,6], the multivariate *t*-distribution was recently adopted due to its potential applications in applied statistics reliability [29]. The PDF of the *n*-variate $t_{n}\left(\mu ;\left[D,G\right];v\right)$-distribution [30] with *v* degrees of freedom around the process and measurement noises is given by:

(2a) $$p_{n}\left(\phi_{k}|D_{k},v,\mu_{k}\right)=\frac{\Gamma \left(\left(n+v\right)/2\right)}{\Gamma \left(v/2\right){\left(\pi v\right)}^{n/2}{\left|D\right|}^{1/2}}{\left[1+\frac{1}{v}{\left(\phi_{k}-\mu_{k}\right)}^{T}{D_{k}}^{-1}\left(\phi_{k}-\mu_{k}\right)\right]}^{-\left(n+v\right)/2}$$

(2b) $$p_{n}\left(\gamma_{k}|G_{k},v,\mu_{k}\right)=\frac{\Gamma \left(\left(n+v\right)/2\right)}{\Gamma \left(v/2\right){\left(\pi v\right)}^{n/2}{\left|G\right|}^{1/2}}{\left[1+\frac{1}{v}{\left(\gamma_{k}-\mu_{k}\right)}^{T}{G_{k}}^{-1}\left(\gamma_{k}-\mu_{k}\right)\right]}^{-\left(n+v\right)/2}$$

where $D_{k}$ and $G_{k}$ are the symmetric/positive definite scale matrices related to the process and measurement noises, respectively, whereas $\mu_{k}\in IR^{n}$ is the location parameter and Γ(⋅) stands for the gamma function. The vector $\mu_{k}$ specifies the location of the single mode of the distribution. The invertible matrices $D_{k}$ and $G_{k}$ indicates the relative width of the central mode along each dimension and also the correlation between dimensions. The covariance matrices associated to the process and measurement random vectors $\phi_{k}$ and $\gamma_{k}$ are, respectively, provided by

(3a) $$R_{k}=\frac{\nu }{\nu -2}D_{k}$$

(3b) $$Q_{k}=\frac{\nu }{\nu -2}G_{k},\;\text{for all}\;\nu >2$$

**Figure 1:**
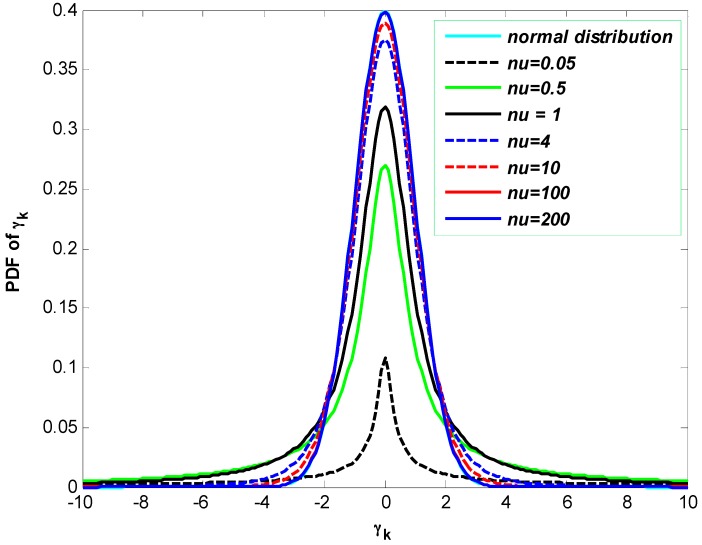
The PDFs of the *t*-distribution for 0.05, 0.5, 1, 4, 10, 100 and 200 degrees of freedom and the PDF of the standard Gaussian distribution (cyan) related to the measurement noise $\gamma_{k}$.

The degree of freedom *v* controls the Student density’s kurtosis (the heaviness of the tails) of the distribution. As illustrated in Figure 1, when the value of degrees of freedom increases, the *t*-distribution approaches the Gaussian distribution with mean 0 and variance 1. The degree of freedom can refer to as the shape parameter, since the peakedness of distribution is decreased or increased by varying *ν* (see Figure 1).

The purpose of this paper is to find approximations to the smoothing distributions $p\left(X_{k}|Y_{1:K}\right)$ for k=1,…,K taking into account the non-Gaussianity of the process and the measurement noises which follow the multivariate *t*-distribution as given by Equations (2a) and (2b).

## 3. Non-Gaussian Delayed Particle Smoothing Method and Computation of the Proposal Distribution Parameters

The basic smoothing problem is the computation of ${\hat{X}}_{k|k^{\prime }}\approx p\left(X_{k}|Y_{k^{\prime }}\right)$ with $k<k^{\prime }$. In a recursive context, the smoothing problems can be divided into three categories: (1) fixed-interval smoothing, where the conditional probability mass function (PMF) of the state given the observation history is provided by $p\left(X_{k}|Y_{1:K}\right)$ for all time indices k=1,…,K; (2) fixed-delay smoothing (called also fixed-lag smoothing), where the density $p\left(X_{k}|Y_{1:k+L}\right)$ is computed online with L>0, the fixed-delay; and (3) the fixed point smoothing, where $p\left(X_{k}|Y_{1:S}\right)$ is computed for a fixed value *k* with the increasing S>k. The fixed-delay smoothing problem can also be considered as the combination of the fixed-point and the fixed-interval smoothing problems. The delayed sampling technique is close to the sequential fixed-interval smoother [31] except that the state vector to be estimated excluding the first and the initialization steps is kept unchanged through time.

### 3.1. Non-Gaussian Delayed Particle Smoothing Method (nGDPS)

The standard sequential importance sampling (SIS) technique can be adapted to achieve the above smoothing problems. The SIS technique consists of recursive propagation of the weights and support points as each measurement is received sequentially [32]. The SIS is responsible of approximating the posterior distribution at time k−1, $p\left(X_{0:k-1}|Y_{1:k-1}\right)$, with a weighted set of samples ${\left\{X_{0:k}^{\left(i\right)},w_{k}^{\left(i\right)}\right\}}_{i=1}^{M}$, also called “particles” in particle filtering (PF) [33] system. In the case of fixed-point particle smoothing (FPPS) and the fixed-interval particle smoothing (FIPS) methods, the posterior distributions at time k−1 with a weighted set of samples ${\left\{X_{1:k}^{\left(i\right)},w_{k}^{\left(i\right)}\right\}}_{i=1}^{M}$ are given by the marginal smoothing density $p\left(X_{k}|Y_{1:k-1}\right)\approx \sum_{i=1}^{M}w_{k-1}^{i}\delta \left(X_{k}-X_{k}^{i}\right)$ and the joint smoothing density $p\left(X_{1:k-1}|Y_{1:k-1}\right)\approx \sum_{i=1}^{M}w_{k-1}^{i}\delta \left(X_{k-1}-X_{k-1}^{i}\right)$, respectively. However, the FPPS method suffers from the employed fixed-point smoother, which only updates the most recent estimate when a delayed measurement occurs [34]. Similarly, due to the resampling step, for large interval value, the approximation to the smoothing distribution related to the FIPS method turns out to be strongly depleted and inaccurate [20].

The *a posteriori* joint PMF from the fixed-delay particle smoothing [21] is defined by $p\left(X_{1:k-1}|Y_{1:k+L-1}\right)\approx \sum_{i=1}^{M}w_{k-1}^{i}\delta \left(X_{k-1}-X_{k-1}^{i}\right)$ where $X_{k-1}^{i}$ is the sample (particle) generated from an importance distribution $q\left(X_{1:k-1}|Y_{1:k+L-1}\right)$ at time k−1, $X_{1:k-1}$ represents the system states and $Y_{1:k+L-1}$ the observations. To solve this, we exploit a delayed particle smoothing (DPS) method with the purpose of achieving the optimal filtering. Moreover, by taking into account the process and measurement noises that are assumed to be non-Gaussian distributed, we propose a novel framework of non-Gaussian delayed particle smoother (nGDPS) for the application of vehicle localization and state estimation. From the perspective of optimal filtering, where a constant delay is tolerated in the estimates, the proposed nGDPS method is able to provide the accurate approximation to the smoothing distribution when the delay time is small.

In the following subsection, we will derive the computation of the proposal distribution parameters for nGDPS. The detail of the design of nGDPS method is presented in Section 4.

### 3.2. Computation of the Proposal Distribution Parameters for nGDPS

The choice of an appropriate form of proposal distribution, which represents the true posterior density, is an important step in the particle smoothing since it can reduce the number of samples required for a certain level of performance such as the generation of the accurate estimates [35]. In practice, the proposal distribution, which is the crucial feature of any particle filter, is defined as close to the final posterior as possible, *i.e.*, $q\left(X_{k}^{i}|X_{k-1}^{i},Y_{k}\right)\approx p\left(X_{k}^{i}|X_{k-1}^{i}\right)$. However, the challenge in the selection of proposal distribution is to find an appropriate covariance matrix for the random walk. In general, it is difficult to design such a proposal since it produces the variance of likelihood distribution, which is usually relatively small, and this leads to the inaccurate approximation of the target distribution [36]. Moreover, in the majority of cases, nonlinearity or non-Gaussianity makes an analytic solution intractable [37].

In this paper, alternatively, the importance density is assumed as the multivariate normal N(μ,Γ), where *μ* denotes the mean and Γ is covariance matrix [38]. The objective is to design a strategy to handle the estimate mean $\hat{\mu }={\hat{X}}_{k|k-1}^{i}$ and covariance matrix $\hat{\Gamma }={\hat{P}}_{k|k}^{i}$ of the proposal distribution for nGDPS. To achieve this, we propose a two-fold approach, which consists of two parts: (1) design an approach based on Ensemble KF (EnKF) for computing the mean and covariance of the proposal distribution; and (2) propose a delayed Gibbs sampling method to cope with the degeneracy problem of nGDPS.

#### 3.2.1. EnKF Approach for Computing the Mean and Covariance of the Proposal Distribution

Kalman Filter (KF) and its variants such unscented Kalman Filter (UKF) and Extended Kalman Filter (EKF) [39] have been widely used to compute the mean and covariance of proposal distribution. However, the main drawbacks of these methods are based on the fact that the densities of the process noise $\phi_{k}$ and measurement noise $\gamma_{k}$ are constrained to be Gaussian.

To compute the proposal distribution parameters when noises are non-Gaussian distributed, we design a computation method based on Ensemble Kalman Filter (EnKF) to deal with the mean and the covariance matrix of the proposal non-Gaussian distribution. The mean ${\hat{X}}_{k|k-1}^{i}$ and covariance matrix ${\hat{P}}_{k|k}^{i}$ of the proposal distribution for each propagated particle $X_{k-1|k-1}^{i}$ are given by:

(4) $${\hat{X}}_{k|k-1}^{i}=\frac{1}{M}\sum_{i=1}^{M}X_{k|k-1}^{i}$$

(5) $${\hat{P}}_{k|k}^{i}=\frac{1}{M-1}\sum_{i=1}^{M}\left[\psi_{k}^{i}\right]{\left[\psi_{k}^{i}\right]}^{T}$$

(6) $$\psi_{k}^{i}=X_{k|k}^{i}-{\hat{X}}_{k|k}^{i}$$

The Ensemble Kalman gain is then provided by:

(7) $$\Sigma_{k}^{j}=\frac{\sum_{i=1}^{M}\left[a_{k}^{i}\right]{\left[b_{k}^{i}\right]}^{T}}{\sum_{i=1}^{M}\left[b_{k}^{i}\right]{\left[b_{k}^{i}\right]}^{T}}$$

where $a_{k}^{i}=X_{k|k-1}^{i}-{\hat{X}}_{k|k-1}^{i}$ and $b_{k}^{i}=h\left[X_{k|k-1}^{i},\gamma_{k}^{i}\right]-\frac{1}{M}\sum_{i=1}^{M}h\left[X_{k|k-1}^{i},\gamma_{k}^{i}\right]$. Based on Equations (4) and (5), the importance density can be given by:

(8) $$q\left(X_{k}^{i}|X_{k-1}^{i},Y_{k}\right)\approx p\left(X_{k}^{i}|X_{k-1}^{i}\right)=N\left({\hat{X}}_{k|k-1}^{i},{\hat{P}}_{k|k}^{i}\right)$$

This is then used to draw at time k=0 a number *β* of samples from the initial state ${\hat{X}}_{0|0}^{i}$ as follows:

(9) $$X_{0|0}^{i}={\hat{X}}_{0|0}^{i}+{\left[{\hat{P}}_{0|0}^{i}\right]}^{\frac{1}{2}}\varphi^{i},\;\varphi^{i}\sim N\left(0,I\right)$$

where the associated initial weight is set to $w_{0}^{i}=1/\beta$.

Moreover, at each instant *k* step, the proposal distribution Equation (8) is utilized to draw a sample around each particle based on the following equation:

(10) $$X_{k|k}^{i}={\hat{X}}_{k|k}^{i}+{\left[{\hat{P}}_{k|k}^{i}\right]}^{\frac{1}{2}}\varphi^{i},\;\varphi^{i}\sim N\left(0,I\right)$$

The weights $w_{k}^{i}$ associated with each particle at each instant *k* are recursively defined by $w_{k}^{i}\propto w_{k-1}^{i}\frac{p\left(Y_{k}|X_{k}^{i}\right)p\left(X_{k}^{i}|X_{k-1}^{i}\right)}{q\left(X_{k}^{i}|X_{k-1}^{i},Y_{k}\right)}$. Therefore, based on Equation (8), we have:

(11) $$w_{k}^{i}\propto w_{k-1}^{i}\frac{p\left(Y_{k}|X_{k}^{i}\right)p\left(X_{k}^{i}|X_{k-1}^{i}\right)}{N\left(X_{k}^{i};{\hat{X}}_{k|k-1}^{i},{\hat{P}}_{k|k}^{i}\right)}$$

At each measurement instant, every particle weight is re-initialized to 1/M in the case where a resampling scheme is applied methodically [25], thus Equation (11) can be given by:

(12) $$w_{k}^{i}\propto \frac{p\left(Y_{k}|X_{k}^{i}\right)p\left(X_{k}^{i}|X_{k-1}^{i}\right)}{N\left(X_{k}^{i};{\hat{X}}_{k|k-1}^{i},{\hat{P}}_{k|k}^{i}\right)}$$

Furthermore, based on EnKF contribution in particle filtering, the proposal distribution and the smoothing weight, in context of delayed particle smoothing (DPS) are provided by Equations (13) and (14), respectively:

(13) $$q\left(X_{k+l}^{i}|X_{k+l-1}^{i},Y_{k+l}\right)\approx N\left({\hat{X}}_{k|k+l-1}^{i},{\hat{P}}_{k|k+l}^{i}\right)$$

(14) $$w_{k}^{i}\propto \frac{p\left(Y_{k+l}|X_{k+l}^{i}\right)p\left(X_{k+l}^{i}|X_{k-1}^{i}\right)}{N\left(X_{k}^{i}-{\hat{X}}_{k|k+l-1}^{i};{\hat{X}}_{k|k+l-1}^{i},{\hat{P}}_{k|k+l}^{i}\right)}$$

where ${\hat{P}}_{k|k+l}^{i}=\frac{1}{M-1}\sum_{i=1}^{M}\left[\psi_{k+l}^{i}\right]{\left[\psi_{k+l}^{i}\right]}^{T}$ is the smoothing covariance, and ${\hat{X}}_{k|k+l-1}^{i}=\frac{1}{M}\sum_{i=1}^{M}X_{k|k+l-1}^{i}$ represents the smoothing mean provided by the EnKF such that $\sum_{i=1}^{M}w_{k+l}^{i}=1$ for l=0,…,L.

#### 3.2.2. Delayed Gibbs Sampling Method for Degeneracy Problem of nGDPS

Although it is easy to draw sample from the Gaussian proposal distribution and to perform the weight update, the variance of the importance weights can only increase over time and this technique suffers from the degeneracy problem. It means that particles with weak normalized weights are discarded and particles associated to strong weights are at duplicated. To avoid such degeneracy of the particles, different resampling strategies have been developed [40,41]. In this paper, we design an integrated method, in which the resampling method incorporates smoothing of the sampled trajectories over a fixed delay.

For each particle $X_{k}^{i},i=1,\ldots ,M$, $X_{k+L}^{i}$ is sampled based on Markov Chain Monte Carlo (MCMC) kernel Θ such that $X_{k+L}^{i}\sim \Theta \left(X_{k+L}^{i}\right)$ where *L* is the fixed delay. More details on MCMC resampling approach and the choice of its kernels can be found in [42]. Instead of selecting the next state all at once, we adapt the Gibbs sampling method [43] for sampling from the joint MCMC kernel, where a separate probabilistic choice for each of the *m* dimensions is made and each choice is up to the other m−1 dimensions. A similar method has also been applied on simultaneous localization and mapping (SLAM) [44] by performing single move at each time step. Note that $\Theta \left(X_{k+L}^{i}\right)$ can be obtained using the definition of conditional probability as follows [45]:

(15) $$\Theta \left(X_{k+L}^{i}\right)=p\left(X^{i}|X_{k+L}^{1},\ldots ,X_{k+L}^{i-1},X_{k+L-1}^{i+1},\ldots ,X_{k+L-1}^{M}\right)=\frac{p\left(X^{i}|X_{k+L}^{1},\ldots ,X_{k+L}^{i-1},X_{k+L-1}^{i},X_{k+L-1}^{i+1},\ldots ,X_{k+L-1}^{M}\right)}{p\left(X_{k+L}^{1},\ldots ,X_{k+L}^{i-1},X_{k+L-1}^{i+1},\ldots ,X_{k+L-1}^{M}\right)}$$

where $X^{i}$ is a collection of all particles, *i.e.*, $X^{i}={\left\{X^{i}\right\}}_{i=1}^{M}$. During this process, new particles are used as soon as they are obtained.

## 4. nGDPS Algorithm for Vehicle Localization

**Figure 2:**
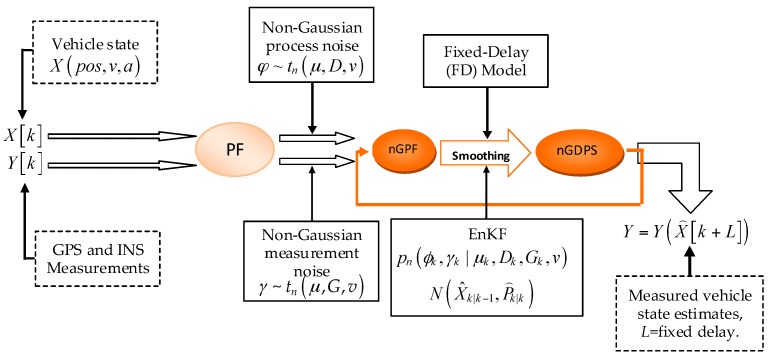
The architecture of the proposed method.

The proposed nGDPS supposes that the non-Gaussian process and measurement noises follow the multivariate *t*-distribution. The architecture of the proposed nonlinear approach is summarized in Figure 2. This nonlinear/non-Gaussian method relies on smoothing technique where fixed-delay and EnKF models are deliberately integrated in particle filter (PF) aiming to estimate the vehicle state. The state vector of vehicle is denoted by $X_{k}={\left[pos_{k},v_{k},a_{k}\right]}^{T}$, where $pos_{k}$, $v_{k}$ and $a_{k}$ are the position, velocity, and acceleration, respectively. The state of the subset of particles around the vehicle state $X_{k+L}$ at a given time is determined where *L* represents the fixed-delay size. Moreover, this algorithm refers to the resampling technique, which incorporates smoothing of the sampled trajectories over a fixed delay based on Gibbs sampling method, aiming to successfully avoid the degeneracy of particles. Algorithm 1 shows the pseudo-code implementing the proposed non-Gaussian delayed particle smoother (nGDPS) for vehicle localization approach.

**Algorithm 1** Non-Gaussian Delayed Particle Smoother (nGDPS) for Vehicle Localization***% Initialization:***At time k=0:Set $X_{0}={\left[pos_{0},v_{0},a_{0}\right]}^{\prime }$, the state vector representing the initial information of vehicle where $pos_{0}$ , $v_{0}$, and $a_{0}$ are position, velocity, and acceleration respectively;Select initial covariance matrices $R_{0}$ and $Q_{0}$ related to the measurement and process noises respectively;Draw *M* particles and set the weight $w_{0}\left(X_{0}^{i}\right)=\frac{1}{M}$, i=1,⋯,M given that the prior knowledge $X_{0}^{i}\sim p\left(X_{0}^{i}\right)$;Set the fixed-delay size *L* and sample time *K*.**For** each time instant k=1,…,K
**do****For** each particle i=1,…,M
**do**
*% **Importance sampling*****:**Compute the state of Λ particles where Λ is the subset of particles around the vehicle state $X_{k+L}$ at time k+L (*i.e.*, Λ⊆M);${\left\{X_{k+L-1}^{i}\right\}}_{j=1}^{\Lambda }\leftarrow X_{k+L}^{j,i}\sim q_{EnKF}\left(X_{k+L}^{j,i},X_{k+L}^{i}|X_{k+L-1}^{i},Y_{k+L}\right)$, where $q_{EnKF}\left(\cdot \right)$ stands for the importance function defined using EnKF according to Equation (13);**For** each l=0,…,L **do**
Update the process and measurement noise densities based on Equations (2a) and (2b)The definite scale matrices in terms of the associated covariance matrices are given by $D_{k+l}^{i}=\frac{\nu -2}{\nu }R_{k+l}^{i}$ and $G_{k+l}^{i}=\frac{\nu -2}{\nu }Q_{k+l}^{i}$, (ν≠0) respectively.% **Importance weight update:**Compute new weight according to Equation (14);
**End For** ***% Normalization:*** The normalized weight is given by ${\tilde{w}}_{k+L}^{i}\leftarrow w_{k+L}^{i}*{\left(\sum_{j=1}^{\Lambda }w_{k+L}^{j}\right)}^{-1}$, and the state of the vehicle at time *k* + *L* is provided by: ${\overset{⌣}{X}}_{k+L}\leftarrow {\overset{⌣}{X}}_{k+L}+X_{k+L}^{i}*{\tilde{w}}_{k+L}^{i}$.***% Resampling:*** Compute the effective sample size ${\hat{\lambda }}_{e}$ as defined in [46];**If** ${\hat{\lambda }}_{e}<\bar{\lambda }=\frac{2\Lambda }{3}$ (the predefined threshold) **then**Resample using the Delayed Gibbs sampling (DGS) method, otherwise,
***% Output the smoothed estimation*** Compute the smoothed state estimates of the vehicle: ${\overset{⌢}{X}}_{k+L}$
**End If**  **End For****End For**


## 5. Experimental Results and Analysis

### 5.1. Simulation Setup

**Figure 3:**
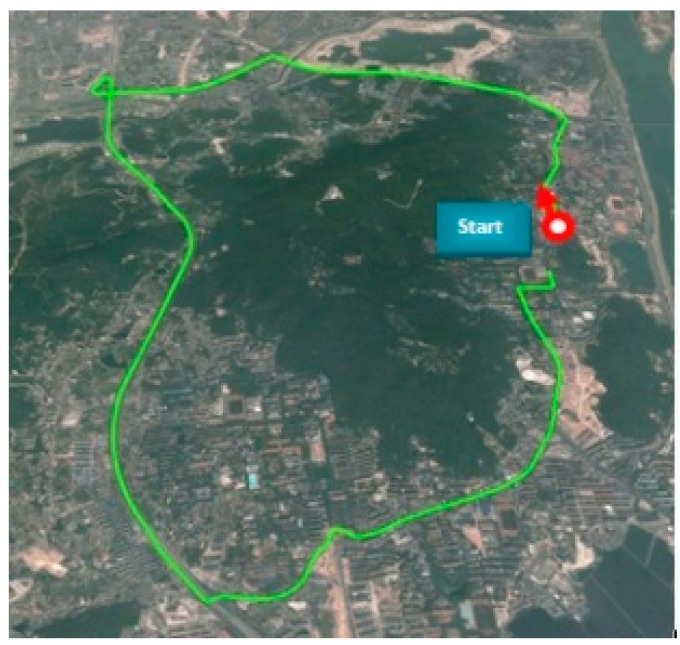
Test site and path (original photo from Google Map).

In order to evaluate its performance, the proposed framework of nGDPS is applied to a vehicle state estimation problem. The test vehicle was driven along different roads in Hunan University area with different velocities for about 25 min. During the road experiment, the open air environment and urban area are included. Figure 3 shows the vehicle trajectory from the satellite map, which was used for the evaluation.

In this experiment, the test vehicle is equipped with several low-cost sensors, *i.e.*, GPS receiver, wheel speed sensor, Gyroscope and acceleration sensors, which provide the vehicle state information such as position, velocity, and acceleration. The measurement accuracy of sensors is summarized in Table 1. Note that data from different satellites in view were collected using only A-GPS during the road experiments. This new advanced technology based on mobile computing system has gained a large popularity nowadays in various applications such as in target localization [22,47], tracking systems [48] and vehicle positioning [49].

Several implementations were conducted to study the performance of the proposed approach. To achieve this, we used the real traffic measurement data collected using the technique explained above. At each time *k*, valid vehicle information such as position (*pos*), angular velocity (*v*) and linear acceleration (*a*) provided by GPS receiver, wheel speed, gyroscope and accelerometer sensors. Although these sensors provide vehicle information in three-dimension system, that is, $pos\left(p_{k}^{x},p_{k}^{y},p_{k}^{z}\right)$, $v\left(v_{k}^{x},v_{k}^{y},v_{k}^{z}\right)$ and $a\left(a_{k}^{x},a_{k}^{y},a_{k}^{z}\right)$ expressed in terms of East-North-Up (ENU) coordinate system, a two-dimension system (East-North) is considered in this paper for simplicity. Hence, the state vector is composed of six elements such that $X_{k}=\left[p_{k}^{x},\;p_{k}^{y},\;v_{k}^{x},\;v_{k}^{y},\;a_{k}^{x},\;a_{k}^{y}\right]$. The position range $p_{k}^{x}$ was between 112.909 and 112.946 deg, whereas $p_{k}^{y}$ varied from 28.164 to 28.208 deg. The angular velocities $v_{k}^{x}$ ranged from −3.535 to 3.658 deg/s and $v_{k}^{y}$ from −1.561 to 3.500 deg/s. Moreover, the accelerations $a_{k}^{x}$ and $a_{k}^{y}$ varied from −2.714 to 2.044 m/s^2^ and from −3.424 to 2.388 m/s^2^, respectively. In addition, the test was started with the following initial values $X_{0}^{j}=\left[112.942,\;28.187,\;0.030,\;0.020,\;0.022,\;0.070\right]$, which represent, respectively, the initial position (deg), velocity (deg/s), and acceleration (m/s^2^) in North (or X) and East (or Y) coordinates.

### 5.2. Comparison and Analysis Results

For our simulations, different parameters have been chosen as follows: the fixed delay is set to L=3 [11], whereas the number of particles is 1000 in all adopted methods and due to the presence of random variables, the times of Monte Carlo runs are set to 100. The total duration of the experiment is K=1410 s. The degrees of freedom parameter is set to v=4, whereas the state dimension is m=6. Moreover, the initial covariance matrices related to the process and measurement noises are set to $R_{0}={\left[0.1,\;0.1,\;0.1,\;0.1,\;0.1,\;0.1\right]}^{T}$ and $Q_{0}=diag\left[1,\;1,\;1,\;1,\;1,\;1\right]$, respectively. We would set $R_{0}$ to zero and this assumes there is no initialization error and our initial states are perfect, which is not the case. Then we set to $0.1*I_{6}$ where $I_{6}$ is the Identity matrix. The choice of the initial measurement noise covariance $Q_{0}$ (bigger than $R_{0}$, $1*I_{6}$) is due to the fact that this can make the convergence fast and hence the state estimate errors become negligible. With the intention of accelerating the convergence of the EnKF and therefore improving the accuracy of the sample covariance, the size of ensemble is set to β=100 [24]. Then, in order to highlight the performance of our proposed method, we first evaluate the estimation improvement based on the non-Gaussianity of the noises, by introducing the so-called Gaussian delayed particle smoother (GDPS) where process and measurement noises are assumed to be Gaussian distributed. As illustrated in Figure 1, we get the (univariate) Gaussian distribution from the multivariate *t*-distribution $\phi_{k}\sim t_{n}\left(\mu_{k};G_{k};v\right)$ and its PDF is computed based on Equation (2) by setting m=1, the mean μ=0 and the degree of freedom ν=200 [50].

**Figure 4:**
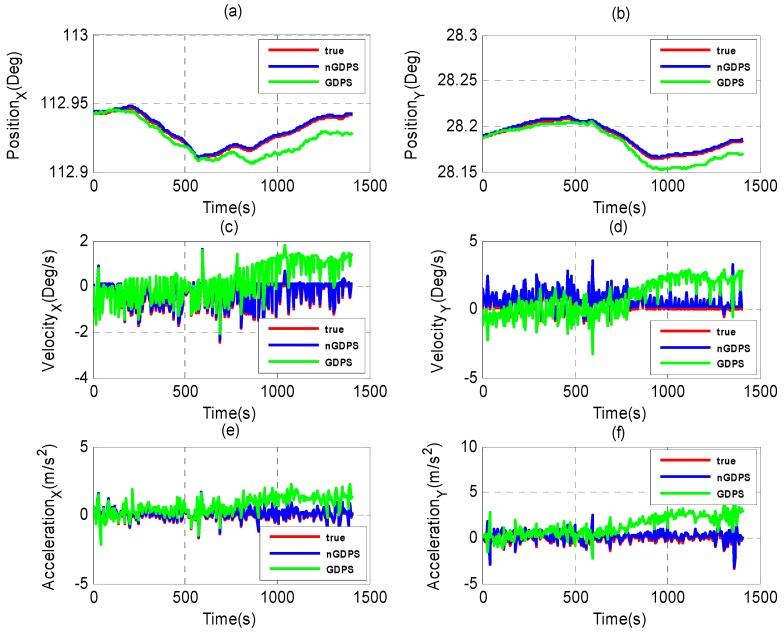
Estimate results of nGDPS and GDPS *versus* true vehicle state in (X, Y)-coordinates: (**a**,**b**) Position; (**c**,**d**) Velocity; and (**e**,**f**) Acceleration.

The performance of nGDPS and GDPS for the state vehicle estimation is depicted in Figure 4. From Figure 4, it can be observed that GDPS is less accurate than nGDPS. By setting the small value of degree of freedom, v=4, for nGDPS (comparing to GDPS where v=200), this decreases not only the scale matrices defined in terms of the weighting covariance matrices but also the PDFs related the process and measurement noises during the update step of our proposed method. Therefore, the vehicle state estimation is improved.

We then evaluate the influence of non-Gaussianity model by comparing our proposed method with the Delayed Particle Smoother where both process and measurement noises are assumed to be Gaussian distributed, and this is referred to GDPS method. In this experiment, we obtain the Gaussian measurement distribution from the multivariate *t*-distribution $\phi_{k}\sim t_{n}\left(\mu_{k};G_{k};v\right)$ and its PDF is computed based on Equation (2) by setting n=1, the mean μ=0 and the degree of freedom tends to infinity (v→+∞). In this scenario, the process noise for both nGDPS-VSE and GDPS follow the standard normal distribution, *i.e.*, $\left(\lambda_{k},\gamma_{k}\right)\sim N\left(0;G=Q\right)$ where *Q* is the covariance matrix related to the process noise $\lambda_{k}$.

**Figure 5:**
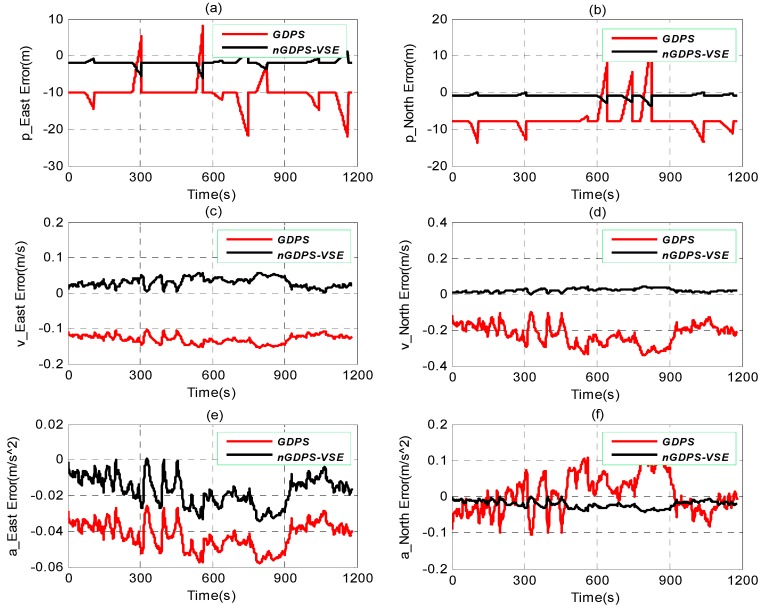
Vehicle state estimate (VSE) errors plot depicting the performance of GDPS, nGDPS-VSE through the influence of the non-Gaussianity related to the measurement noise. Position estimate: (**a**) East and (**b**) North. Velocity estimate: (**c**) East and (**d**) North. Acceleration estimate: (**e**) East and (**f**) North.

From Figure 5, the proposed nGDPS where measurement noise is multivariate *t*-distributed outperforms GDPS where measurement noise is normally distributed in terms of vehicle state accuracy. In fact, the curve of GDPS is far away from the line Y≡y=0 comparing to the proposed nGDPS for vehicle state estimate (VSE). The main reasons are due to the drawbacks of the regular Gaussian measurement model, which diverged during the experiments.

Therefore, the choice of multivariate *t*-distribution to deal with the measurement noise as well as the EnKF to handle the non-Gaussian PDF plays a great role in estimation of vehicle state. In addition, the increased position errors in Figure 5a,b are due to the GPS outages detected during the road experiments.

**Figure 6:**
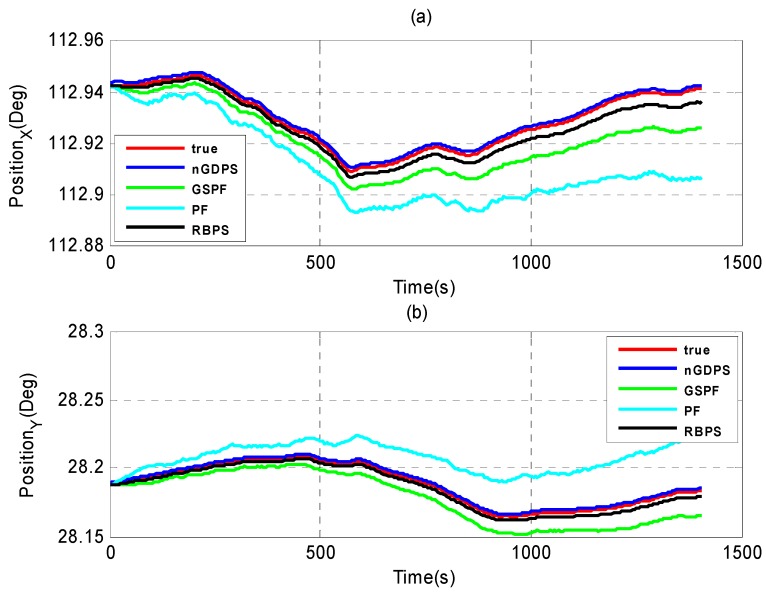
Estimate position results of nGDPS, GSPF and RBPS *versus* true vehicle state in (X, Y)-coordinates. (**a**) X-coordinates; (**b**) Y-coordinates.

**Figure 7:**
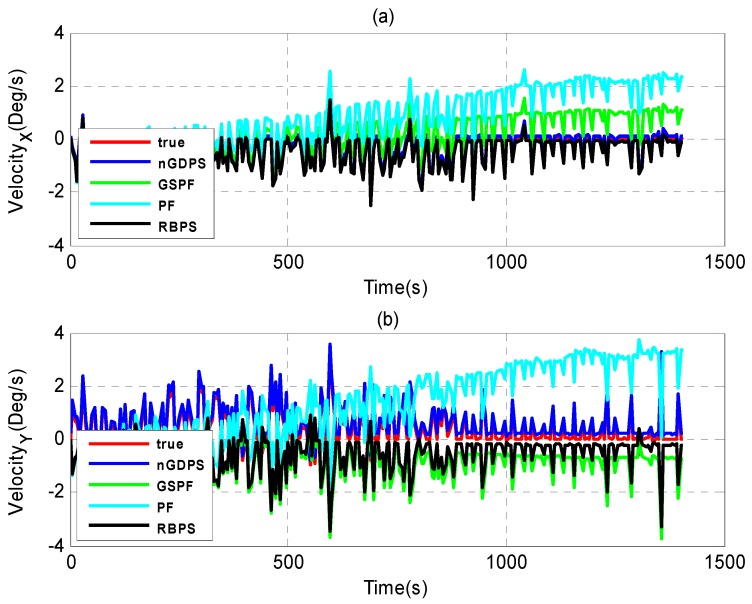
Estimate velocity results of nGDPS, GSPF and RBPS *versus* true vehicle state in (X, Y)-coordinates. (**a**) X-coordinates; (**b**) Y-coordinates.

**Figure 8:**
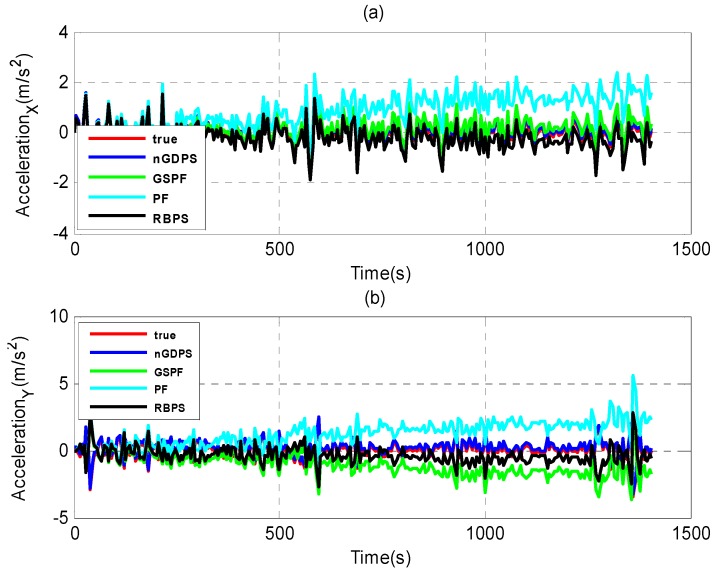
Estimate acceleration results of nGDPS, GSPF and RBPS *versus* true vehicle state in (X, Y)-coordinates. (**a**) X-coordinates; (**b**) Y-coordinates.

Furthermore, we evaluate the performance of our proposed nGDPS compared to two types of estimation methods: (1) the filtering methods such as Gaussian Sum Particle Filtering (GSPF) [8] and the standard PF [33]; and (2) the smoothing method such as Rao-Blackwellized particle smoother (RBPS) [21], as illustrated in Figure 6, Figure 7 and Figure 8.

As can be seen in Figure 6, Figure 7 and Figure 8, although GSPF can overcome degeneracy and has good performance for the state estimation in non-Gaussian noise, the proposed nGDPS is more accurate than GSPF. This is due to the fact that the conditioning on the history of measurements in our proposed method is expected to bring the smoothed estimates closer to the true vehicle state than filtering alone. On the other hand, although RBPS and nGDPS are both based on particle smoothing method, it is noticeable that RBPS is less accurate than nGDPS. The reason behind this is the way the error covariance in the PDFs approximation of the process and measurement noises are handled and the usage of EnKF in the nGDPS method that is used to deal with the mean and covariance of proposal distribution.

Moreover, instead of considering only the past and current observations, the fixed-delayed formulation takes also into account the future observation and performs the estimate of the state of the system. As a result, it is able to provide the accurate approximation to the smoothing distribution in general. The vehicle localization accuracy is also augmented by the behavior of the considered Gibbs sampling method, which avoids the weight degeneracy problem during the particle smoothing by incorporating smoothing of the sampled trajectories over the fixed delay, *L*. The use of this resampling method is also motivated by the fact that in its form, resampling is useful in evaluating conditional estimate errors particularly in a non-Gaussian environment as supported by authors in [51].

Finally, to evaluate the performance of our proposed method, the statistical analysis based on the root mean squared errors (RMSE) metric was also used. Table 2 summarizes the results, in terms of the time averaged RMSE values for different estimation methods based on filtering and smoothing techniques. Note the low RMSE implies the high confidence for the vehicle localization and state estimation methods. As expected, in Table 2, the proposed nGDPS method gives the most accurate results when compared with the previously considered estimation methods, for instance, the fixed-point particle smoothing (FPPS) and of fixed-interval particle smoothing (FIPS).

The large RMSE values for PF, GSPF and RBPS are particularly a result of the linearization errors in computing the state covariance matrices. In addition, the standard PF and GSPF are relatively less accurate in terms of RMSE comparing to other smoothing methods (RBPS, FIPS, FPPS, GDPS and nGDPS) due to its shortcomings associated to the filtering problems such as the fact that filtering methods in their basic form only calculate the estimates of the current state of the system given the history of measurements which may affect the vehicle state accuracy. Therefore, given the adequate PDF estimators (multivariate *t*-distribution, in our case), the proposed method, which uses smoothing technique and considers noises as non-Gaussian distributed, can significantly decreases the root-mean-squared state errors.

## 6. Conclusions and Future Work

In this paper, a novel nonlinear framework on smoothing method, non-Gaussian delayed particle smoother (nGDPS), is proposed, which takes into account the non-Gaussian noisy environments that are assumed to follow the multivariate *t*-distribution. To improve the estimation accuracy of the standard particle filter, the fixed-delay technique is integrated into the proposed nGDPS to cope with measurement information and the error covariance of the system. The delayed Gibbs sampling method, which incorporates smoothing of the sampled trajectories over a fixed delay, is designed to handle the degeneracy of particles. Moreover, a computation approach based on EnKF is proposed with the purpose of dealing successfully with the proposal distribution parameters when noises are non-Gaussian distributed. The performance of the proposed approach is validated with respect to real-world data collected using low-cost on-board vehicle sensors in urban environment. Both the experiments and the statistical analysis demonstrate the effectiveness of the proposed nGDPS, which can significantly improve the vehicle state accuracy and outperform the existing filtering and smoothing methods. Future work will be focused on the application of the proposed approach on the real-world data collected in more challenging environment. The core issue in PF in general, and in the proposed smoothing approach, is the reduction in computation time, which will be also be addressed in the future work.

## Figures and Tables

**Table 1:** Measurement Accuracy of sensors.

Sensors	Type	Range
GPS	BCM 4752	Doppler: 0.03 m/s, Pseudo range: 0.50 m, Carrier phase: 0.05 m
Gyroscope	MPU 6500	±250/500/1000 deg/s
Accelerometer	MPU 6500	±2 g/4 g/8 g
WSS	-	0.05 m/s

**Table 2:** RMSEs (m) for the X and Y coordinates averaged over 1000 runs: state errors with respect to the true vehicle state (position, velocity and acceleration).

Methods	Pos (X)	Pos (Y)	Velocity (X)	Velocity (Y)	Acc (X)	Acc (Y)
PF	1.0586	1.1544	31.8801	22.8954	28.1493	41.9005
GSPF	0.2732	0.2800	28.0227	22.5702	27.5700	35.9537
RBPS	0.1844	0.1501	12.2739	20.0224	18.2701	27.3539
FIPS	0.1182	0.1320	10.3371	14.7937	12.9007	19.5511
FPPS	0.0908	0.0774	7.7410	12.8522	5.9010	13.8574
GDPS	0.0556	0.0473	1.3790	9.8386	3.3093	9.0792
nGDPS	0.0508	0.0715	3.3791	7.1948	3.3795	7.1344
